# Research of Pathogenesis and Novel Therapeutics in Arthritis 3.0

**DOI:** 10.3390/ijms241210166

**Published:** 2023-06-15

**Authors:** Chih-Hsin Tang

**Affiliations:** 1 Department of Pharmacology, School of Medicine, China Medical University, Taichung 404333, Taiwan; chtang@mail.cmu.edu.tw; Tel.: +886-2205-2121 (ext. 7726); Fax: +886-4-2233-3641; 2 Chinese Medicine Research Center, China Medical University, Taichung 404333, Taiwan; 3 Department of Biotechnology, College of Health Science, Asia University, Taichung 400354, Taiwan; 4 Department of Medical Research, China Medical University Hsinchu Hospital, Hsinchu 30272, Taiwan

Arthritis has a high prevalence globally and includes over 100 types, the most common of which are rheumatoid arthritis (RA), osteoarthritis (OA), psoriatic arthritis (PsA), and inflammatory arthritis. All types of arthritis share common features of the disease, including monocyte infiltration, inflammation, synovial swelling, pannus formation, stiffness in the joints, and articular cartilage destruction. The exact etiology of arthritis remains unclear, and no cure exists as of yet. Anti-inflammatory drugs (NSAIDs and corticosteroids) are commonly used in the treatment of arthritis. However, these drugs are associated with significant side effects, such as gastric bleeding and an increased risk for heart attack and other cardiovascular problems. It is therefore crucial that we continue to research the pathogenesis of arthritis and seek to discover novel modes of therapy.

Our call for papers for this Special Issue attracted several articles, all of which underwent rigorous peer review. A total of 18 papers (13 research papers and 5 reviews) satisfied the inclusion criteria for publication in this Special Issue. The research papers cover cellular, preclinical, and clinical investigations. The reviews discuss aspects of treatment with hyaluronic acid, corticosteroids, and platelet-rich plasma in patients with temporomandibular joint OA, the potential of a novel micro-immunotherapy medicine that uses ultra-low doses of proinflammatory cytokines as well as other immune factors in an attempt to restore bodily homeostasis in patients with RA, a discussion of vascular endothelial growth factor (VEGF) biology and its potential as a therapeutic target in rheumatic diseases, and lastly, a summary of current insights into the involvement of the CCN family in RA and OA, accompanied by evidence in support of the targeting of CCN proteins in these diseases. All of these papers are discussed below.

(i) Cellular investigations. Sohn and colleagues describe how the synthesis of adenosine by human RA or OA synoviocytes is crucial to anti- or pro-inflammatory effects mediated by adenosine receptor subtypes A2A and A2B in response to interleukin (IL)-6, IL-10, and tumor necrosis factor (TNF) [1]. The researchers suggest how this function may be exploited to serve as a future therapeutic strategy in RA or OA [1]. Immunofluorescence analysis of immunophenotypes of different cell populations in knee synovial tissue samples obtained from healthy controls or patients with early or advanced knee OA helps to elucidate the pathophysiology of this disease, according to Ostojic and colleagues [2]. They found that macrophages appear to be the most active cells in early OA through the nuclear factor-κB (NF-κB) production of inflammatory factors (inducible nitric oxide synthase [iNOS] and matrix metalloproteinase-9 [MMP-9]) in the intima, whereas, in advanced OA, NF-κB is mostly expressed by leukocytes in the synovial subintima (stroma) [2]. Ostojic and colleagues suggest that it may be worth blocking macrophageal and leukocyte NF-κB expression to slow the disease behavior of OA [2]. The other research group indicates that cytokines released in complete Freund’s adjuvant (CFA)-induced arthritis in ICR mice as well as the regulation of blood levels of cytokines by two peptides of the innate immunity protein Tag7 (PGLYRP1) capable of blocking the activation of the TNFR1 receptor [3]. The Taiwanese research group also demonstrates that IL-17 promotes vascular cell adhesion molecule 1 (VCAM-1) production in human OA synovial fibroblasts (OASFs) and subsequently increases monocyte adhesion by reducing miR-5701 expression through the PKC-α and JNK signaling cascades, which may help with the design of more effective OA treatments [4].

(ii) Preclinical investigations. Makalish and colleagues describe how treatment with the antisense oligonucleotide Cytos-11 effectively inhibits TNF-α gene expression in a rat model of RA, with results that are comparable to those from other studies using adalimumab treatment in rats with RA [5]. Makalish and colleagues detail reductions in joint inflammation and pannus development, reduced lymphocytic infiltration of joint tissues, and decreases in peripheral blood concentrations of TNF-α [5]. Sykora and colleagues describe how administering intraperitoneal injections of high-dose unconjugated bilirubin significantly improved the clinical course of disease in rats with adjuvant-induced arthritis [6]. These researchers acknowledge that while murine models of arthritis have many limitations with regard to human RA, the specific effects of unconjugated bilirubin administration in this experimental model of arthritis may be relevant for further RA pharmacotherapy investigations [6]. In experiments involving mice with collagen-induced arthritis (CIA), administration of the natural flavone kurarinone has shown promise as a potentially beneficial adjunct treatment option for RA, according to Tang and colleagues [7]. In particular, kurarinone inhibited pathogenic Th1 and Th17 cell differentiation and exerted antioxidative activity, which contributed to the amelioration of arthritis in the CIA mice [7]. Experimental investigations conducted by Vita and colleagues indicate that berberine may have a role as a prophylactic supplement for RA [8]. They report that in mice with CIA, berberine markedly delayed the onset of arthritic symptom onset, apparently by suppressing T cell populations [8]. Vita and colleagues call for future investigations that examine the effects of berberine on the immune system under normal physiological conditions [8]. The research team from Canada used two metrological properties of two performance-based outcome measures including Effort Path (Path) and Stairs Assay Compliance (Stairs) for feline OA [9]. The results suggest that both are promising performance-based outcome measures to better diagnose and manage feline OA pain [9]. Using the inhibitory peptide sequence IA9 of triggering receptors expressed on myeloid cells (TREMs) are a family of activating immune receptors that regulate the inflammatory response, diminished release of proinflammatory cytokines, and dramatically suppressed joint inflammation and damage in CIA mice [10].

(iii) Clinical investigations. Investigations into serum levels of brain-derived neurotrophic factor (BDNF) have found significantly increased levels in patients with RA, as opposed to low serum levels in RA patients with anxiety, but not depression, or who were using biologic therapy, report Lai and colleagues [11]. They call for further study into the effect of elevated serum BDNF levels in patients with RA or other systemic autoimmune diseases, such as primary Sjögren’s syndrome [11]. In another study, which analyzed the distribution patterns of iNOS, MMP-9, and the anti-apoptotic protein BCL-2 in the hip synovial tissue of patients with OA, the results suggest that iNOS, MMP-9, and BCL-2 expression regulates hip OA [12]. Korchynskyi and colleagues describe a potential glycosylation site at the asparagine-14 residue within human monocyte chemoattractant protein-1 (MCP-1) revealing lower expression levels in mammalian expression systems [13]. The glycosylation-mediated recombinant chemokine stabilization allows the production of citrullinated MCP-1, which can be effectively used to calibrate crucial assays in RA [13].

Reviews. The five reviews in this Special Issue begin with a systematic review conducted by Derwich et al., regarding the current evidence on the mechanisms of action and the efficacy of hyaluronic acid (HA), corticosteroids (CS), and platelet-rich plasma (PRP) in the treatment of patients aged ≥ 16 years with temporomandibular joint (TMJ) OA [14]. The researchers conclude that the evidence is inconclusive; it does not definitively clarify whether such methods of treatment are more beneficial than arthrocentesis alone for patients diagnosed with TMJ OA [14]. The second systematic review and meta-analysis, registered on PROSPERO (CRD42021279368), shows a weak efficacy of collagen and a very marked non-effect of chondroitin-glucosamine nutraceuticals, which recommends that the latter products should no longer be recommended for pain management in canine and feline OA [15]. The other review describes the use of micro-immunotherapy medicine in RA, a holistic therapeutic approach that employs ultra-low doses of IL-1β, IL-2, and TNF-α, as well as other immune factors, to restore bodily homeostasis [16]. Jacques and colleagues summarize the evidence showing how micro-immunotherapy treatment is associated with fewer adverse effects than the currently available single-target approaches designed to inhibit RA inflammation [16]. Van Le and colleagues have reviewed the evidence on VEGF biology and its function in rheumatic diseases, the contribution of VEGF bioavailability in the pathogenesis of rheumatic diseases, and the potential implications of therapeutic approaches targeting VEGF for these diseases [17]. The last review in this Special Issue is by MacDonald and colleagues, who discuss the involvement of the CCN family in the inflammatory pathologies of RA and OA, as well as the potential of the CCN proteins as therapeutic targets in these diseases [18].

Our hope is that this Special Issue will encourage more research into the pathogenesis of arthritis that ultimately leads to new therapeutic strategies.
